# Does GPER1 Play a Role in Sexual Dimorphism?

**DOI:** 10.3389/fendo.2020.595895

**Published:** 2020-10-30

**Authors:** Janine L. Dovey, Nandini Vasudevan

**Affiliations:** School of Biological Sciences, University of Reading, WhiteKnights Campus, Reading, United Kingdom

**Keywords:** social behavior network, estrogen receptor isoforms, sex differences in brain, neuroestrogens, aromatase, mood, behavior

## Abstract

Estrogens are critical in driving sex-typical social behaviours that are ethologically relevant in mammals. This is due to both production of local estrogens and signaling by these ligands, particularly in an interconnected set of nuclei called the social behavioural network (SBN). The SBN is a sexually dimorphic network studied predominantly in rodents that is thought to underlie the display of social behaviour in mammals. Signalling by the predominant endogenous estrogen, 17β-estradiol, can be either *via* the classical genomic or non-classical rapid pathway. In the classical genomic pathway, 17β-estradiol binds the intracellular estrogen receptors (ER) α and β which act as ligand-dependent transcription factors to regulate transcription. In the non-genomic pathway, 17β-estradiol binds a putative plasma membrane ER (mER) such as GPR30/GPER1 to rapidly signal *via* kinases or calcium flux. Though GPER1’s role in sexual dimorphism has been explored to a greater extent in cardiovascular physiology, less is known about its role in the brain. In the last decade, activation of GPER1 has been shown to be important for lordosis and social cognition in females. In this review we will focus on several mechanisms that may contribute to sexually dimorphic behaviors including the colocalization of these estrogen receptors in the SBN, interplay between the signaling pathways activated by these different estrogen receptors, and the role of these receptors in development and the maintenance of the SBN, all of which remain underexplored.

## Introduction

The steroid hormone 17β-estradiol (E_2_) is the most physiologically relevant estrogen, with a myriad of effects that is dependent on signaling from a receptor. The classical genomic mode of estrogen signaling is *via* nuclear estrogen receptors (ER) α and β, which translocate to the nucleus upon ligand binding to act as transcription factors, regulating transcription over hours to days (1). Nongenomic signaling is a second mode of estrogen signaling which employs membrane-limited forms of ERα and ERβ, as well as the novel G protein-coupled estrogen receptor (GPER)1, to activate second messenger pathways resulting in rapid outputs within seconds to minutes. In the brain, E_2_ acts *via* both signaling mechanisms to facilitate spinogenesis and dendrite growth (2, 3), cell survival (4), and neuroprotection (5). All these processes contribute to the sexual differentiation of the brain, a process that is restricted to critical periods of development in conserved nuclei of the brain referred to as the social behavior network [SBN; (6)]. After development, the SBN remains responsive to E_2_ acting *via* the ERs, integrating information about external and internal stimuli to drive sexually dimorphic expression of behaviors including reproductive behaviors, aggression and anxiety, and to some extent neuroprotection. In this review, we detail the contribution of the various ERs to the formation of the sexually dimorphic SBN and to the local production of estrogens, with areas of future exploration highlighted.

## The Social Behavior Network

The social behavior network (SBN) is a conserved set of hypothalamic and limbic nuclei that contribute to the expression of sex-typical social behaviors (6) *via* sexually dimorphic nuclei (SDN). These are structures within the SBN that differ in volume, cell type, and receptor expression between sexes. The neuroanatomical connections, and the contribution of each SBN nuclei to social behavior has been reviewed in detail in (7).

**Figure 1:**
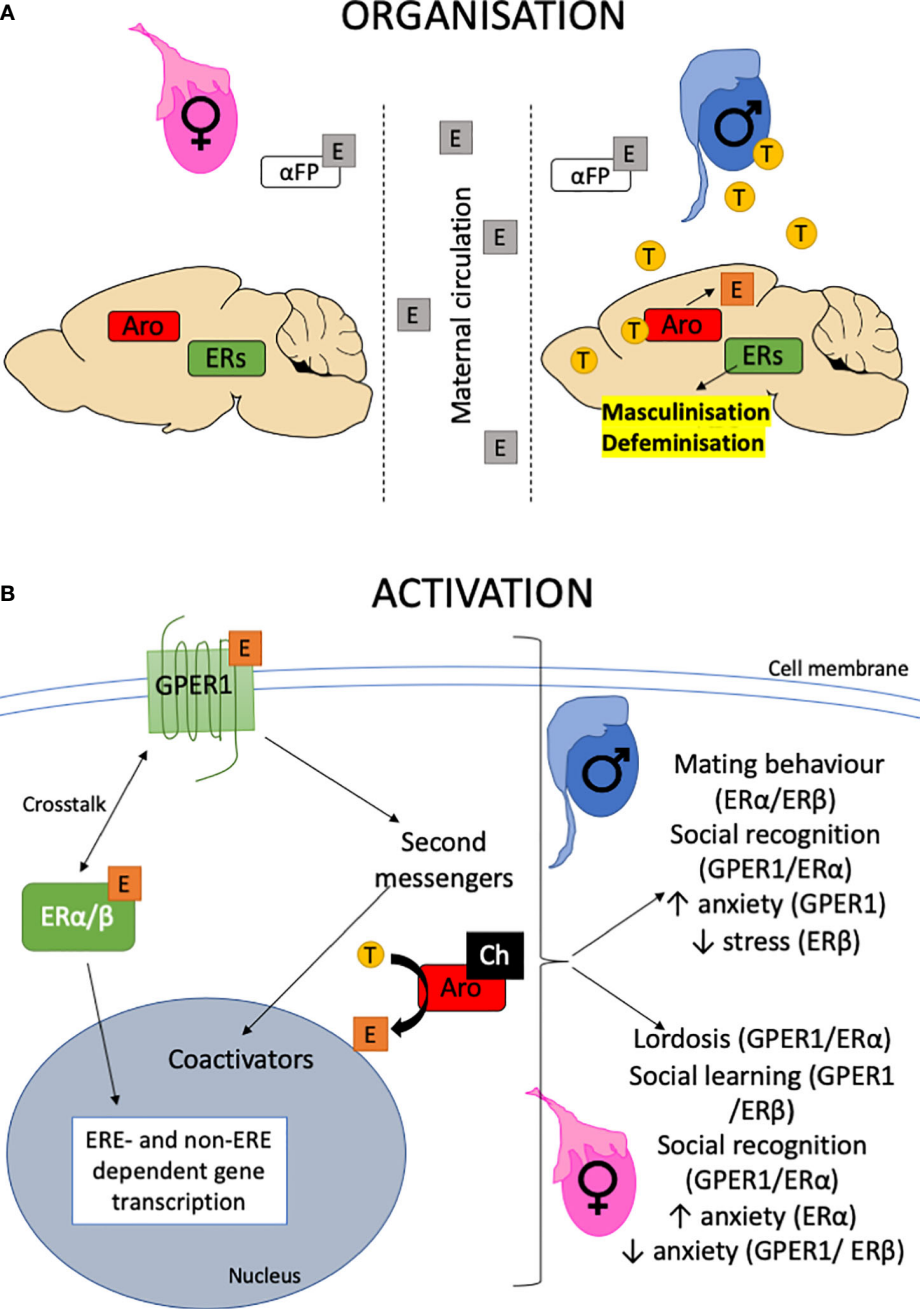
Organizational-activational hypothesis. **(A)** The testes are active during perinatal development providing testosterone for central aromatase (Aro) to produce estrogen (E) within the brain. Estrogen organizes the brain by binding to ERs, leading to the masculinization and defeminization of the brain. By contrast, the perinatal ovary is quiescent. *In utero*, the brain is protected from estrogens that may enter *via* maternal circulation by the presence of α-fetoprotein that binds estrogen. The role of the GPER1 in this organizational period is largely unknown. For a detailed review, the reader is referred to (8) and references therein. **(B)** The organized neural substrate is activated following puberty when the gonads become active. Estrogen is released from the ovaries and testosterone (T) from the testes, which is then aromatized to estrogen in the brain. The availability of cholesterol (Ch) and presence of steroidogenic enzymes within the brain also allows for the *de novo* production of neuroestrogens. Estrogens activate neural circuits to express behaviors through activating second messenger pathways such as MAPK acutely and recruiting transcriptional coactivators such as fos and jun to regulate non-ERE containing promoters. This could be *via* multiple ERs, including GPER1 (9). Alternatively, the classical nuclear hormone receptors, ERα/β can translocate to the nucleus to directly bind estrogen-response-elements in DNA to regulate gene transcription. Both these pathways result in modulation of behaviors in both males and females.

E_2_ in the critical developmental period organizes the SBN (**Figure 1**) *via* molecular mechanisms that include neurogenesis (10, 11), programmed cell death (12), and synaptogenesis (13) and pruning (14). Following reproductive maturation, E_2_ then activates the SBN. Which ERs regulate these processes? In the female hippocampus, both ERα and GPER1 increase spinogenesis *via* ERK and JNK pathways (15) to consolidate spatial memories. Moreover, GPER1 activation leads to rapid increases in hippocampal spine density and promotes social cognition (16, 17). In neocortical cultures, GPER1 activation increases apoptosis mediated by the endocrine disrupter benzoquinone (18) while GPER1 activation can increase the migration of stem cells in the subventricular zone (19). Presumably, these processes are required for GPER1 modulation of sex-typical behaviors such as lordosis and social cognition. For details of the GPER1 including pharmacology, subcellular distribution and signaling, its role in behavior including its modulation of ERα function, the reader is directed to both (9, 20).

### Preoptic Area of the Hypothalamus

The sexually dimorphic nucleus of the preoptic area (SDN-POA) has a larger volume in the male due to increased cell density (21). The perinatal androgen surge at E18 and subsequent aromatization to E_2_ protects dopaminergic cells in the male SDN-POA from apoptosis (22). The receptor for preserving the volume of the SDN-POA is ERα, since WT and androgenized female rats treated with antisense oligonucleotides against ERα show a significantly smaller SDN-POA volume compared to their respective controls (23) though ERα expression levels are not significant between the sexes (**Table 1**). Non-genomic signaling is critical since male mice with a mutation that destroys the tethering of the ERα to the membrane and its ability to initiate non-genomic signaling showed decreased calbindin-immunoreactive (a marker for the SDN-POA) neurones (37). Knockdown of *Gper* in zebrafish resulted in a greater number of cells stained with acridine orange, a marker for apoptosis (38). However, specific brain regions were not identified, and it is not known if this role for GPER1 exists in rodent species. Indeed, the localization of GPER1 within the SDN-POA has not been directly investigated, though efferents from the mPOA to the VTA do express GPER1 (39). Yet, despite its role in non-genomic signaling, the establishment of sexual dimorphisms by GPER1 in the POA is unknown.

**Table 1:** Sexual dimorphisms in central ER and aromatase expression across development.

Area	ERα			ERβ			GPER1			Aromatase		
	Pn	Pb	A	Pn	Pb	A	Pn	Pb	A	Pn	Pb	A
**Hypothalamus**												
ARH	= ^7,8^	= ^7^		X ^3^	= ^3^				= ^13^			= ^11^
VMH	= ^7,8^	F ^7^		F ^6^	= ^6^	=* ^12^			= ^13^	=* ^4^		
PVH		= ^7^				=* ^12^						= ^11^
LS	= ^7^	= ^7^							= ^13^			
AVPV	F ^1,9^		F ^9^	X ^1^	X ^10^				= ^13^			
mPOA	= ^7,8^	F ^7,8^		= ^3^	F ^3^	M* ^12^			= ^13^	X* ^4^		M ^2,11^
**Extended amygdala**												
BNST	F ^1,9^	= ^7,9^	F ^9^	= ^1^		M* ^12^			= ^13^	= ^1^ M* ^4^		M ^2^
MeA		= ^7^				F* ^12^			= ^13^	=* ^4^		M ^2^
**Bird song areas**								M ^5^	M ^5^			

Relative expression of receptors and aromatase during perinatal (Pn), pubertal (Pb), and adult (A) periods. “F” denotes a greater expression in females, “M” a greater expression in males, “=“ an equal expression between males and females, and “X” indicates undetectable expression. All referenced research uses mouse or rat (*) models, apart from one study which used zebra finches to study GPER1 expression in song areas. References 1–5 measured mRNA expression; references 7–12 measured protein expression; reference 6 measured both mRNA and protein. 1. (24). 2. (25). 3. (26). 4. (27). 5. (28). 6. (29). 7. (30). 8. (31). 9. (32). 10. (33). 11. (34). 12. (35). 13. (36).

### Anteroventral Periventricular Nucleus of the Hypothalamus

The anteroventral periventricular nucleus (AVPV) contrasts from the neighboring SDN-POA as females harbor greater cell volumes (40), greater numbers of glia (41), and greater numbers of dopaminergic neurones (40) compared to males. The surge of E_2_ availability in the perinatal male brain upregulates caspase activity and cell death whilst new cells are added to the female AVPV during puberty (11) though the ER that mediates this is not clear.

Adult females express greater amounts of ERα than males (**Table 1**) and levels are not affected by gonadectomy (GDX) (32), which suggests that differences in expression occur prior to adulthood. ERKOα male mice have a greater AVPV volume (24) and a greater number of tyrosine hydroxylase (TH)-positive cells (42) than WT males. However, ERαKO males still have significantly less dopaminergic neurones than WT females (42), suggesting that another receptor contributes to the masculinization of the AVPV. ERβ may be a candidate since it is coexpressed with both ERα and TH in the female AVPV (43) and ERKOβ males have increased TH-ir compared to their WT counterparts (44). Together, this suggests that the sexual dimorphism in dopaminergic cell populations in the AVPV is driven by high levels of E_2_ in the male brain acting through both ERα and ERβ to drive cell death. Interestingly, in cultured dopaminergic neurones shown to express both ERα and GPER1, E_2_ is neuroprotective (5), suggesting that GPER1 may have a modulatory effect on ERα signaling. Though knockout of both α and β ERs (ERKO) has no effect on glial cell numbers in the male AVPV (24), the death of glial cells is an E_2_-dependent process since aromatase KO (ArKO) mice have increased numbers of glial cells (24). This suggests that another ER, such as GPER1, that is abundant in glia, may contribute to the masculinization of the AVPV. Indeed, in an oxygen-glucose deprivation model, GPER1 increases apoptosis of cortical astrocytes (45). Neither expression of the GPER1 protein, nor its colocalization with other ERs in the AVPV, have been characterized in the male or female rodent.

### The Medial and Extended Amygdala

The medial amygdala (MeA) is a major source of input to the medial (m)POA, responsible for relaying olfactory information that underlies social recognition. Similar to the AVPV, new cells are added to the MeA during puberty albeit solely in the male (11). Targeted knockdown of ERα in the MeA in pubertal male mice feminizes the volume of the MeA by reducing neuron numbers (46), suggesting ERα-mediated signaling is important in the establishment of volumetric sex differences.

Aromatase is strongly expressed in the MeA of male mice, particularly in nerve fibers (34) and may contribute to the modulation of synaptic properties of the female MeA across the estrous cycle (47), as E_2_ inhibits neural transmission from the MeA (48). Indeed, administration of the GPER1 agonist G-1 attenuates the upregulation of NMDA receptors in the female basolateral amygdala and blocks the downregulation of GABA_A_ receptors to increase inhibitory synaptic transmission (49).

The bed nucleus of the stria terminalis (BNST) is part of the extended amygdala and plays a key role in stress and anxiety-denoting behaviors (50), expressing both ERα and ERβ during developmental periods (**Table 1**). A subregion of the BNST, the principal nucleus of the BNST (BNSTp) is larger in males than females (51). Administration of testosterone propionate (TP) to females at P1 increases volume, although not to a level comparable with males (52–54). This may be a reflection of greater aromatase expression within the male BNST (34), allowing the brain to generate more estrogen to produce a greater magnitude of masculinization. In addition, it could be a reflection of less androgen receptor (AR) expression in the female brain (55), since masculinization of the BNSTp requires both E_2_ signaling and testosterone signaling *via* the AR (24). Similar to the AVPV, both ERα and ERβ are required for complete masculinization of the BNSTp, since PPT and DPN (ERα and ERβ agonists respectively) given in the perinatal period increase cell number of the female BNSTp, but neither completely mimicked the effects of E_2_ alone (56), suggesting that synergy between ERs, including GPER1 may maintain sexual dimorphism.

The pattern of expression of the ERs (**Table 1**) and the use of pharmacological and genetic studies to target them suggest that the development of the SBN frequently depends on a combination of ERα and GPER1 though it often appears that the role of ERα is predominant. This suggests that both membrane-initiated signaling and classical transcriptional signaling might be important for sex-typical behaviors that are responsive to external stimuli over longer time frames. The idea that GPER1 may facilitate or antagonize ERα signaling has been reviewed in (20) with examples given within and outside the brain. Given that the male brain expresses more aromatase and has more neuroestrogens (Section 3), we speculate that in most instances, neuromorphological organizational changes are driven by these ERs in the male, rather than the female brain.

A number of caveats exist to the localization data. First, most studies have compared the longitudinal dynamics of ER expression in the SBN of wildtype (WT) animals, focusing largely on sexual dimorphisms within one particular age window and/or nucleus. Unusually, a recent study showed that ERα and GPER1 were higher in the striatum of both male and female rats during development and perinatal life but then declined in a sexually dimorphic manner as development proceeded (57); however, they did not explore such developmental dynamics in the SBN. Secondly, due to antibody issues, colocalization studies of GPER1 with the other ERs have not been performed.

## Local Estrogen Synthesis Within the SBN

Apart from the contribution of the ERs, another mechanism that affects SBN nuclei is the provision of local estrogens. The brain expresses the enzymes required to synthesize estrogens *de novo* (neuroestrogens) (58, 59). Developmentally, central aromatase may be important for allowing specific regions to access higher concentrations of E_2_ to maintain cell numbers or drive apoptosis, although more evidence is required to support this idea. In an activational context, aromatase may be important for maintaining stable concentrations of neuroestrogens when systemic concentrations fluctuate across the estrous cycle, as seen in female baboons (60).

### Regulation of Aromatase: Substrate Availability and Development

Is the regulation of aromatase sexually dimorphic? In limbic areas, aromatase activity appears to be constitutive (61). Therefore, the regulation of aromatase activity is proposed to rely on two different systems: a gonad-sensitive hypothalamic system and a non-gonad-sensitive limbic system (62, 63). Though there are no sex differences in aromatase mRNA expression in the BNST or AVPV during perinatal development (24), male rodents have greater levels of aromatase gene expression than females by adulthood (25). In line with this, prepubertal GDX in males reduces aromatase activity in adulthood (64), suggesting that aromatase expression is pubertally organized by pubertal gonadal hormones.

The regulation of central aromatase may also be determined by estrogens themselves. In MCF-7 cells, aromatase activity is upregulated by estrogens in a positive autocrine feedback loop *via* either ERα or GPER1 (65, 66). In transgenic mice that express EGFP in aromatase-positive neurons, EGFP is more highly co-expressed with ERα, ERβ and AR in the male BNST and MeA than in the adult female though co-expression of the ERs and AR with EGFP was prevalent in other nuclei of the SBN of both sexes (34). In contrast, aromatase is mostly co-expressed with ERα during the perinatal period (67), highlighting the potential to investigate developmental change in co-expression, which may be partly explained by the sexually dimorphic addition of new cells during puberty (11). How GPER1 regulates aromatase in the SBN is a question that is currently being investigated by us.

## Discussion: Therapeutic Potential for GPER1

Why is the contribution of GPER1 to a sexually dimorphic SBN important? A sexually dimorphic brain results in sexually dimorphic disorders that are important to consider clinically. For example, neurodegenerative diseases disproportionately affect women (68), whereas learning difficulties such as those associated with autism spectrum disorder and dyslexia are more commonly observed in males (69). Females have a greater risk of developing depression, anxiety, or panic disorders (70) which are correlated with hormonal changes in puberty and menopause (71). E_2_ can elicit anxiogenic or anxiolytic effects in the amygdala (72). ERα knockdown in the medial posterodorsal amygdala (MePDA) resulted in female rats spending more time in the light chamber in the light-dark test (LDT), implicating ERα as anxiogenic (73). On the other hand, there is a general consensus that ERβ is anxiolytic (72) while the role of GPER1 is less clear. Chronic administration of G-1 was anxiolytic in the open field test (OFT), but not the elevated plus maze (EPM) (74) in females while acute administration of G1 was anxiolytic in the EPM within 30 min of administration in males but not females (75). On the contrary, another study found that agonism of GPER1 produced anxiogenic effects in both the OFT and EPM (76) in male and female mice. In this study, G-1 was injected 2h before behavioral testing. Thus, the timeframe of administration may be an important factor in determining the roles of ERs in anxiety. The actions of GPER1 may also depend on the context of anxiety, i.e. whether the animal is previously stressed. Acute stress (imposed by restraint or forced swim tests) significantly decreased the time spent in the open arms and central area of the EPM, but this is ameliorated with G-1 treatment (49) in ovariectomized females. Moreover, acute stress significantly increased the levels of GluR1-containing AMPA receptors and NR2A-containing NMDA receptors, thus increasing small excitatory postsynaptic currents (sEPSCs). However, G-1 treatment reversed these effects, enhancing small inhibitory postsynaptic currents (sIPSCs) instead (49). Thus, GPER1 may be important in mitigating stress-induced anxiety, with little-to-no role in inhibiting behaviors that denote anxiety in the absence of stress. This specificity might allow for the development of personalized medications for anxiety. Furthermore, targeting GPER1 over ERα or ERβ may be preferable given the possible sexual dimorphism in anxiety modulation (75), involvement of the classical ERs in reproductive development and function, and the role of ERβ the in estrogenic modulation of GnRH (77)

## Future Perspectives

Clearly, understanding how GPER1 functions both independently and as a putative modulator of classical ERs in both sexes is imperative for uncovering its therapeutic potential in hormone-associated mood disorders. GPCRs such as the serotonin 1A receptor have been associated with the development of depression. SSRIs function by desensitizing serotonin 1A receptor signaling to decrease plasma levels of oxytocin and adrenocorticotropic hormone (ACTH) (78). The efficacy of SSRIs in attenuating serotonin 1A receptor mediated signaling and consequent oxytocin and ACTH release can be accelerated with G-1 treatment. Dual treatment targeting GPER1 means that symptoms of depression can be alleviated earlier, as it takes up to 12 weeks to reach clinical efficacy with SSRIs alone (79). Furthermore, a recent study has implicated GPER1 as a diagnostic tool for GAD and MDD. Drug-naïve patients with anxiety or depressive disorders exhibit increased serum levels of GPER1, which correlate with anxiety scores (80). This result was found to be independent of sex although mouse models suggest that the role of GPER1 in regulating anxiety is slightly more pronounced in males (76, 81). Though there is a general lack of sexual dimorphism in GPER1 expression with moderate to high distribution of GPER1 in the adult SBN (36), a recent study has shown that GPER1 concentrations decrease with approaching adulthood and the distribution shifts from multicompartment to predominantly cytoplasmic or membrane distribution in the striatum (57). This suggests that GPER1 expression is capable of being developmentally regulated though the significance of such regulation remains unknown. Moreover, the effects of GPER1 activation in adulthood on molecular mechanisms linked to sexual dimorphism raise the possibility that GPER1 may have similar effects in the perinatal and pubertal critical periods. This could be investigated by determining a) the expression of GPER1 in development *versus* adulthood in the SBN and its colocalization with ERα, ERβ; and aromatase; b) the effect of GPER1 agonism with G-1 and antagonism with specific antagonist G-15 and G-36 during the critical periods on sex differences in morphology, neuroestrogen production, and molecular signaling prevalent in the SBN; c) the nature of modulation of ERα action in the SBN. Some of this may be explored with the use of a conditional, regional GPER1KO model, though this is yet to be generated. Therefore, the distinct roles of GPER1 within specific limbic *vs* SBN nuclei in adulthood *versus* developmental periods need to be better understood to produce a targeted medication to alter mood without changing the expression of sex-typical organized behaviors involving GPER1, such as reproduction.

## Author Contributions

Both JD and NV wrote and modified the text of the article after discussions. JD was responsible for the figure and the **Table 1** (which was further modified by NV). All authors contributed to the article and approved the submitted version.

## Funding

JD is supported by the School of Biological Sciences, University of Reading.

## Conflict of Interest

The authors declare that the research was conducted in the absence of any commercial or financial relationships that could be construed as a potential conflict of interest.
